# Exploring the Emerging Evolution Trends of Urban Resilience Research by Scientometric Analysis

**DOI:** 10.3390/ijerph15102181

**Published:** 2018-10-06

**Authors:** Liang Wang, Xiaolong Xue, Yuanxin Zhang, Xiaowei Luo

**Affiliations:** 1 School of Management, Harbin Institute of Technology, Harbin 150001, China; 14b910008@hit.edu.cn; 2 School of Management, Guangzhou University, Guangzhou 510006, China; yuanxin@gzhu.edu.cn; 3 Department of Architecture and Civil Engineering, City University of Hong Kong, Hong Kong 999077, China; xiaowluo@cityu.edu.hk

**Keywords:** urban resilience, CiteSpace, Web of Science (WOS), evolution trends, scientometrics, visualization

## Abstract

Numerous studies in urban resilience have been published in the past decade. However, only a few publications have tracked the evolution trends of urban resilience research, the findings of which can serve as a useful guide for scholars to foresee worth-effort research areas and make the best use of precious time and resources. In order to fill the research gap, this study performed a scientometric analysis on the evolution trends of urban resilience research using a versatile software package-CiteSpace. The scientomentric analysis focuses on distribution of lead authors and their institutions, high frequency categories and keywords, high influential journals, author contribution, and evolutionary trends based on co-author analysis, co-word analysis, co-citation analysis and cluster analysis of documents. This study discoveries that first, the U.S., England, Australia, Canada, China and Sweden are the countries that make the most significant contributions in the advancement of urban resilience research; second, the existing urban resilience research focuses primarily on environmental studies, geography and planning development; third, hot topics of the urban resilience research keep shifting from 1993 to 2016; fourth, the knowledge body of urban resilience research consists of five clusters: resilience exploratory analysis, disaster resilience, urban resilience, urban resilience practice, and social-ecological systems; last, the emerging trends in urban resilience research include defining urban resilience, adaptation model, case studies, analytical methods and urban social-ecological systems, resulting in cutting-edge research areas in urban resilience.

## 1. Introduction

The concept of resilience has become a very popular area of research in terms of publications [1,2,3] and been adopted in various disciplines, such as physics, psychology, and social-ecological systems [4,5,6]. The urban population increased from 10% to over 50% in the past two decades with the unprecedented urbanization process [7]. Resilience is becoming attractive in urban research with cities theorized as highly complex and adaptive systems [8,9]. Urban resilience reflects the ability of urban systems to maintain and rapidly recover to the desired status after external disturbance, adapting to changes and quickly transforming systems to a new balanced status [10]. Despite great popularity among researchers, urban resilience has no commonly accepted definition:

“Urban resilience reflects the degree to which cities tolerate alteration before reorganizing around a new set of structures and processes [11].”

“A disaster-resilient city can be understood as a city: (a) reduce or avoid current and future hazards; (b) reduce current and future susceptibility to hazards; (c) establish functioning mechanisms and structures for disaster response; and (d) establish functioning mechanisms and structures for disaster recovery [12].”

“A climate-resilient city has the capacity to withstand climate change stresses, to respond effectively to climate-related hazards, and to recover quickly from residual negative impacts [13].”

“Urban resilience refers to the ability of an urban system-and all its constituent socio-ecological and socio-technical networks across temporal and spatial scales-to maintain or rapidly return to desired functions in the face of a disturbance, to adapt to change, and to quickly transform systems that limit current or future adaptive capacity [10].”

What is in common among the various definitions is that “urban resilience” is considered a positive trait contributing to the sustainable development of cities. Urban resilience is widely accepted as the ability not only to maintain basic functions, but also to improve the original status of cities [14,15]. Resilience is a positive concept to be an excellent pathway for sustainable development in previous urban studies. However, people have contradictory views in implementation of urban resilience [16,17]. Policy makers focus on prioritizing different resilience agendas [18]. Meanwhile, urban resilience reflects the ability to return to a normal or stable state after disturbance [19,20]. It is difficult to determining what is or is not a desirable normal state, which needs normative judgments to find urban resilient state accepted widely [5,18,21,22,23].

Some new discoveries regarding urban resilience have been proposed and adopted in recent years [24,25,26]. Some researchers explored the complexity of urban resilience from the perspective of complex systems, especially in networked scenarios, where cities were treated as complex and dynamic multisystem consisting of dynamic links between different components [8,27,28,29,30,31,32,33]. In addition, many papers published in renowned journals are case studies in urban studies [34,35,36] and focused on new themes, such as pathways to urban resilience [29,37,38,39,40], urban resilience policies [41,42], urban water resilience [43,44], urban energy resilience [45,46], resilience trade-offs [38], urban form resilience [47], the spatial-temporal characteristics of urban resilience [48,49,50], the impacts of urban resilience on sustainable development [51,52,53] and urban resilience assessment approaches [54,55,56,57,58,59,60,61]. All of these achievements laid a solid foundation for the future urban resilience research.

Although many urban resilience studies have been conducted, few have focused on the evolutionary trends of urban resilience. Some studies in this field systematically analyzed urban resilience from six conceptual tensions: definition of “urban”, understanding of system equilibrium, positive and negative conceptualizations of resilience, mechanisms for system change, adaptation and general adaptability, and timescale of action [10] and have reviewed the mechanisms of micro-resilience of creative urban economy in a context of transition [62]. In addition, some publications have given the potentiality of “Ecological Land Use Supplement” (ELC) biodiversity benefits by integrating information from the publications on urban ecological restoration [63] and summarized the empirical knowledge of urban resilience using case studies [64,65]. However, a common problem of these studies is that the results depended on subjective or empirical judgment. The research results are not universally applicable in different urban resilience scenarios.

It is difficult for new researchers to distinguish major academic journals and core publications from huge urban resilience publications. Thus, a scientometric analysis is needed to explore the evolution trends of urban resilience because it can systematically review related studies. For instance, Fröhlich and Hassink have used scientometric analysis to tackle the issue of fuzziness and stretching concerning regional resilience [66]. Song et al. use similar method to review emerging trends in global PPP (Public-Private Partnership) research [67]. Zhao et al. also use scientometrics to review green building research from 2000 to 2016 [68].

In order to overcome the shortcomings of previous reviews of urban resilience, this study identified the evolution trends of urban resilience research from January 1993 to December 2016 by scientometric analysis. The research results will help global researchers to better understand research status and identify cutting-edge research areas in the field of urban resilience. This paper described the application of the CiteSpace package, the retrieval strategy for data collection, the parameter design for scientific indicator analysis, and then analyze the results obtained using the CiteSpace package. The data visualization software package-CiteSpace is used to show the research status of urban resilience, find the research hot topics in the retrieved publications, and explore the further evolution trends of urban resilience research.

This paper is organized as follows. First, the urban resilience literature is reviewed using traditional method, the shortcomings of previous reviews of urban resilience is also identified in this section. Then, we describe the application of CiteSpace software package, the paper retrieval strategy is proposed for data gathering, and parameters of the scientometric analysis are setup. In results analysis, we attempt to discover the most productive contributors and reveal the major categories and primary research topics in the domain of urban resilience. The distribution of core articles, authors and journals related to urban resilience is also expounded by analyzing co-citation networks. In discussion, we compare the distinctions and relationships between our research results and previous studies. Finally, the hot research topics and development trends in the domain of urban resilience are summarized in the conclusion section.

## 2. Methodology

### 2.1. Introduction to Scientometrics

De Solla Price and Garfield are considered the founding fathers of scientometrics. The latter created the Science Citation Index and initiated the Institute for Scientific Information, which were heavily used for scientometric analysis [69]. The industrialization of science increased the number of publications and novel findings. The advancement of the computing technologies allowed effective analysis of this data [70]. Scientometrics focused on the analysis of publications and was referred to as the scientific and empirical study of science and its outcomes [71]. Scientometric methods include qualitative, quantitative and computational approaches, which concentrate on institutional productivity comparisons, institutional research rankings, journal rankings establishing faculty productivity and tenure standards, assessing the influence of top scholarly articles, and developing profiles of top authors and institutions in terms of research performance [72,73,74,75].

Previous studies use a single scientometric indicator to analyze the research status of different disciplines. Thus, the research results only reflect the research status of disciplines from a specific aspect and cannot really reveal the research status of disciplines from global perspective. This study adopts three types of scientometric indicators. These three types of scientometric indicators are summarized by Cobo et al., including co-author analysis, co-word analysis and co-citation analysis [76]. Co-author analysis analyzes the authors and their affiliations to study the social structure and collaboration networks [77]. Co-word analysis uses the most important words or keywords of the documents to study the conceptual structure of a research field [78]. Co-citation analysis is used to analyze the academic structure of documents that cite the same references; the results of co-citation analysis can be used for cluster analysis [76].

### 2.2. CiteSpace Software Parameters Setting

**Figure 1:**
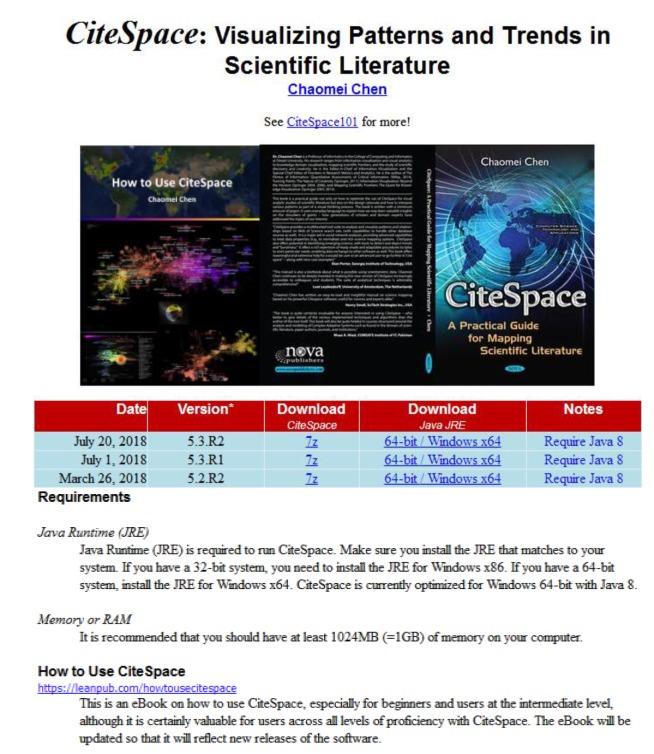
Download interface of CiteSpace software.

The CiteSpace software is important analysis and visualization tool in scientometrics domain. CiteSpace was designed by Chen for progressive knowledge domain visualization [79]. It focused on finding critical points in the development of academic domains, especially intellectual turning points and pivotal points [80]. The latest version of CiteSpace is 5.3.R2 SE for 64-bit Windows with Java 8, and the software download home page was optimized on 20 July 2018 [81]. As free software, CiteSpace software can be downloaded from the website (http://cluster.ischool.drexel.edu/~cchen/ citespace/download/). The download interface of CiteSpace software is shown in Figure 1.

Based on the research trends and themes of Scientific Metris analysis, CiteSpace has different parameter settings and choices, resulting in specific differences in the results of scientific indicators. In this study, the number of networks is the same as the number of years within the time limit of the time scaling value parameter of 1 [80]. Pathfinder was selected as the pruning method, which is applied only to prune the merged network [82,83]. We propose a modular Q value and an average contour value greater than 0.5 to ensure the reliability and importance of the cluster [84,85]. The betweenness centrality between nodes is defined as the shortest paths between other vertex pairs passing through the node [86,87,88]. Moreover, the publications records contain different scientometric informations, such as authors, institutions, keywords, articles and terms, which represent different types of nodes in scientific collaboration networks. In the using process of CiteSpace, users can select a single node type or multiple concurrent node types. The created links represent the relationship between different nodes, which represent co-citation or co-occurrence relationships.

### 2.3. Data Collection for Scientometric Analysis and Visualization

This study adopts the scientometric perspective to review the emerging trends of urban resilience research in urban studies. Three search criteria are established for publication retrieval. Firstly, only academic journals were selected for scientometric analysis, in consideration of their impact positions in urban resilience domain. Secondly, different keywords related to urban resilience are used in the publication retrieval. Thirdly, the time span of publication retrieval should be as long as possible. Based on the above three document retrieval principles, this study searched 39 journals related to the Web of Science (WOS) core collection database for urban studies [89,90]. All of the keywords used in our search strategy were selected based on previous urban resilience studies. Ultimately, three keywords are selected in this study include resilience, resilient and resiliency. The time span of publication retrieval is from January 1993 and December 2016, because the first paper in 39 journals related to urban studies is published in 1993 [91].

After pre-analysis and comparison, we decided to apply the following retrieval strategy: [(keywords = (“resilience” OR “resilient” OR “resiliency”) AND Publication name = (“Landscape and Urban Planning” OR “Journal of Urban Planning and Development” OR “Journal of Urban Economics” OR “European Urban and Regional Studies” OR “Cities” OR “Habitat International” OR “Urban Forestry & Urban Greening” OR “Urban Studies” OR “International Journal of Urban and Regional Research” OR “Journal of Planning Literature” OR “Housing Policy Debate” OR “Environment and Urbanization” OR “Urban Geography” OR “Housing Studies” OR “Journal of Urban Technology” OR “Journal of the American Planning Association” OR “City & Community” OR “European Planning Studies” OR “Journal of Planning Education and Research” OR “Journal of Housing Economics” OR “Housing Theory & Society” OR “Regional Science and Urban Economic” OR “Economic Development Quarterly” OR “Journal of Contemporary Ethnography” OR “Urban Affairs Review” OR “International Regional Science Review” OR “Urban Policy and Research” OR “Journal of Urban Affairs” OR “Real Estate Economics” OR “Urban Education” OR “Journal of Housing and the Built Environment” OR “Journal of Real Estate Finance and Economics” OR “Education and Urban Society” OR “Journal of Architectural and Planning Research” OR “EURE-Revista Latinoamericana De Estudios Urbano Regionales” OR “Urban Design International” OR “Journal of Urban History” OR “Open House International” OR “Urban Lawyer”))]. 405 records are obtained after the publication retrieval process on 18 February 2017, and these records are downloaded and saved in TXT format.

**Figure 2:**
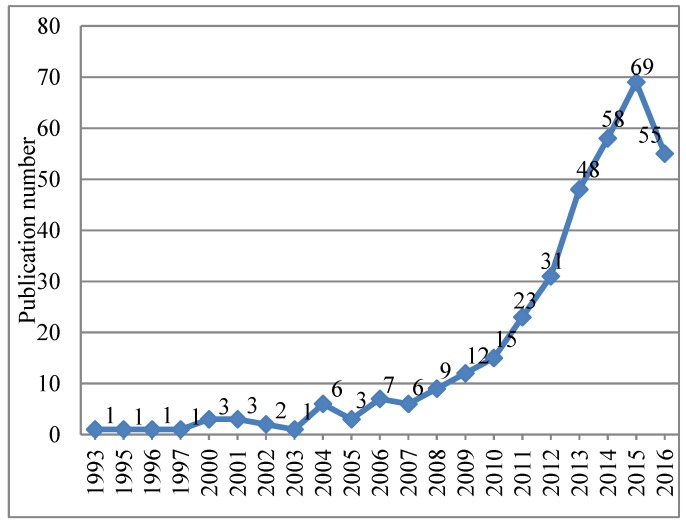
Publications records related to urban resilience in the WOS core collection, published from January 1993 and December 2016.

In the WOS core collection, the publication retrieval records can be analyzed from different aspects, such as author, source journal, category, and references. After eliminating the invalid records, 355 valid document records were obtained, of which includes articles for 96.62% (343) including proceedings papers for 3.1% (11), other 3.38% (12) publications records are reviews. After the pre-analysis, 355 articles and review publications records were selected as the database used in this study during scientometric analysis. The number of these 355 articles published each year is shown in Figure 2.

Figure 2 shows the current state of urban resilience research and shows the distribution of 355 document records for 39 journals related to urban studies in the WOS core database from January 1, 1993 to December 31, 2016. The publication number per year related to urban resilience presents discontinuous characteristic and only has a very small number before 2000. Then, the total number of annual publications related to urban resilience in the WOS presents slight increasing and fluctuation trend between 2000 and 2010. Since then, it has grown steadily since 2010, and the number of publications reached its peak in 2015. Figure 2 details the annual number of publication on urban resilience research.

## 3. Results Analysis

In this section, we analyze a series of scientometric networks using the publications data retrieved in the section of data collection. The relevant publication retrieval records are analyzed and visualized from three aspects: co-author analysis, co-word analysis, and co-citation analysis. Through co-author analysis, we assess and analyze the academic influences of authors, countries and institutions in the field of urban resilience. The main research topics of urban resilience are discovered by co-word analysis. Additionally, the scientific structure and development trends for urban resilience are extracted and generalized by co-citation networks analysis.

### 3.1. Co-Author Analysis

In this section, we analyze the academic cooperation information of authors obtained from the publication retrieval records and assess leading authors, countries, and institutions on urban resilience studies. The scientometric data is also used to generate and analyze the co-author network and the author’s countries and institutional networks.

#### 3.1.1. Co-Authorship Network

**Figure 3:**
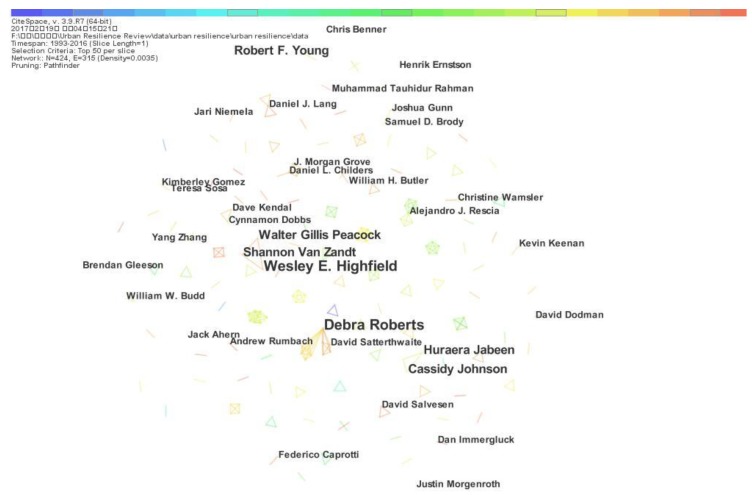
Co-authorship network.

The academic collaboration can describe the productivity level and contribution of researchers to scientific research. Through co-authorship analysis, Figure 3 shows that the author’s contribution on urban resilience research presents a decentralized trend in the co-authorship network; no scholar has an absolute advantage on the publications outputs of urban resilience. The co-authorship network has 424 nodes and 315 links, including the lead authors of publications. The node represents the author, and the link between the two nodes indicates that the two authors directly collaborate through writing papers together.

Figure 3 reveals the outputs and co-author relationships of leading authors in urban resilience research. The size of the authors’ nodes presents a positive correlation to the numbers of authors’ publications. The thicknesses of lines between authors indicate the levels of the collaborative relationships in a given year. The different colors of links represent different phases during January 1993 to December 2016. Figure 3 shows that the most productive authors in the domain of urban resilience research are Wesley E. Highfield, Associate Professor at the Department of Marine Sciences at Texas A&M University and Deborah Roberts, Professor at the Department of Environmental Planning & Climate Protection at EThekwini Municipal, followed by Shannon Van Zandt, Walter Gillis Peacock, Robert F. Young, Huraera Jabeen and Cassidy Johnson. Table 1 lists the authors who have most publications on urban resilience research in recent years.

The co-author relationships only reflect the outputs and contribution of publications on urban resilience research. The highly productive authors are not necessarily highly influential to urban resilience research. The levels of authors’ influence are reflected by co-citation analysis. To solve this problem, the co-citation frequency of authors is compared and analyzed in later co-citation networks analysis.

#### 3.1.2. Network of Co-Authors’ Countries and Institutions

**Figure 4:**
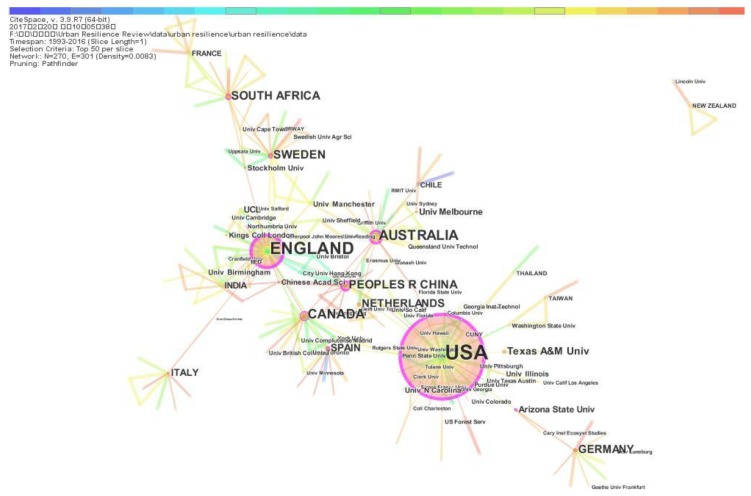
Collaborative relationship network.

The authors’ countries and institutions form a collaborative relationship network which reflects the collaborative relationship between countries and institutions. To characterize the collaborative relationship of publications on urban resilience, CiteSpace is used to form this collaborative relationship network. According to the publication frequency, nodes represent different countries and institutions in collaborative relationship network, the links represent the collaborative relationship between different countries and institutions on urban resilience research. The academic collaborative relationships between countries and institutions on urban resilience research are shown in Figure 4.

Figure 4 reveals collaborative relationships between countries and institutions on urban resilience research. Figure 4 shows that the majority of the total publication output mainly comes from the USA, England, Australia, Canada, China and Sweden. Regarding the geographical distribution of the publications output, the country with the largest publications output is the USA with 136, followed by England with 55, Australia with 24, Canada with 19, China with 15, Sweden with 15, South Africa with 13, Germany with 12, Netherlands with 11 and Spain with 10. The urban resilience research is conducted mainly in countries with high urbanization levels or in the process of urbanization.

Figure 4 shows that the U.S., England and Australia are the first countries to research urban resilience. Although USA is the largest publication contributor among all of the countries, England has almost the same influence as the USA in the collaborative relationship network. Interestingly, some countries that appear as major publication contributors have less influence in the collaborative relationship network, such as The Netherlands. Additionally, China as the biggest country in the process of urbanization has close collaborative relationships with almost countries contributing high publications output, including the USA, England, Australia and Canada.

The lines and circular of nodes in Figure 4 show the contribution and cooperation of research institutions in the field of urban resilience. The USA has the most active research institutions on urban resilience research, with the largest number of publications published between January 1993 and December 2016. The institution with highest number of publications is Texas A&M University (with 10 publications), followed by Arizona State University (6), UCL (6), University of Melbourne (6), King’s College London (5), Stockholm University (5), University of Manchester (5), Chinese Academy of Sciences (4), University of Birmingham (4), University of Illinois (4) and University of North Carolina (4). The top 11 institutions includes four institutions from the USA, other institutions are mainly from countries with high publication outputs, such as England, Australia and China. Stockholm University is ta traditional resilience research center which has some world-renowned researchers on resilience, such as Professor Carl Folke [91].

Betweenness centrality between nodes represents the impact of nodes on the flow of information between other nodes. There are some nodes with high betweenness centrality values that countries such as the USA (0.57), England (0.57), China (0.29), Australia (0.26), Sweden (0.2), Spain (0.2), South Africa (0.15), Canada (0.12), Germany (0.09), Italy (0.07), Netherlands (0.05) and institutions such as Arizona State University (USA, 0.1), University of Manchester (England, 0.08), Gran Sasso Science Institute (Italy, 0.08), Stockholm University (Sweden, 0.07), Florida State University (USA, 0.05). These nodes play important roles in the cooperative network of urban resilience research. Therefore, the publications from countries and institutions with high betweenness centrality should be paid more attentions, including countries such as the USA, England, China, Australia, and institutions such as Arizona State University, University of Manchester, Gran Sasso Science Institute, Stockholm University, Florida State University.

### 3.2. Co-Word Analysis

In this section, we analyze the changes of research topics on urban resilience research from January 1993 to December 2016. The main contents include co-occurring categories network analysis and co-keywords network analyses, which are used to analyze the evolution trends and academic frontiers of urban resilience research.

#### 3.2.1. Network of Co-Occurring Categories

The 355 records retrieved from the 39 journals related to urban research in the WOS Core Collection Database can be attributed to one or more categories based on the WOS categories of source journals. To analyze the subject categories distribution of publications on urban resilience research, we summarize the subject categories of 355 publications on urban resilience research in Table 2, the time period is divided into three panels according to the snapshot of urban resilience publications: 1993–1999, 2000–2010 and 2011–2016. Because all publications are retrieved from journals belonged to urban studies, the category of all 355 publications is Urban Studies. In addition to the subject category of Urban Studies, Environmental Studies (219, 61.69%) has the most abundant publication records, followed by other main categories: Geography (112, 31.55%), Planning Development (95, 26.76%), Geography Physical (70, 19.72%) and Ecology (70, 19.72%).

The number of publications in each category reflects the development trends of urban resilience research in different domains. It is apparent that the main categories include Urban Studies, Environmental Studies, Geography, Planning Development, Geography Physical and Ecology. The numbers of publications in the categories of Urban Studies and Environmental Studies significantly increase during 1993 and 2016, whereas the numbers of publications in the other categories of Geography, Planning Development, Geography Physical and Ecology gradually increase.

**Figure 5:**
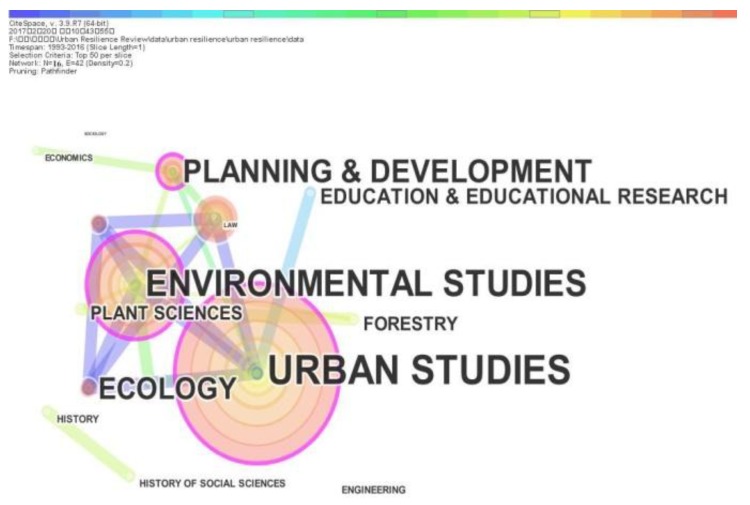
Co-occurring subject categories network.

In addition to the category of Planning Development, all the 5 main categories have the publications of urban resilience in three panels between 1993 and 2016. Other 10 categories contribute the less numbers of publications on urban resilience research during 1993 and 2016. Only the category of Education Educational Research appears in the early research of urban resilience. The three categories of Engineering Civil, Law and Sociology just appear in the research of urban resilience after 2010. In order to accurately analyze the evolution trends of urban resilience, we use CiteSpace to generate the network of co-occurring categories on urban resilience as shown in Figure 5. Figure 5 is co-occurring categories network including 16 nodes and 54 links.

Figure 5 shows that the top three categories in urban resilience research are Urban Studies, Environmental Studies and Ecology. In addition to the subject category of Urban Studies, Environmental Studies is the biggest subject category, which means that urban resilience is mainly researched from the environmental perspective. Interestingly, some categories as the major publication contributors have less influence in co-occurring categories network, such as Ecology and Geography Physical. In Figure 5, the node with the highest betweenness centrality is represented by red-ring. Some nodes with high betweenness centrality in co-occurring subject categories network are Urban Studies (0.17), Environmental Studies (0.15) and Planning Development (0.11), which represent these subject categories are the major turning nodes linking the urban resilience research in different phases and play important roles in the development of urban resilience research.

#### 3.2.2. Network of Co-Occurring Keywords

**Figure 6:**
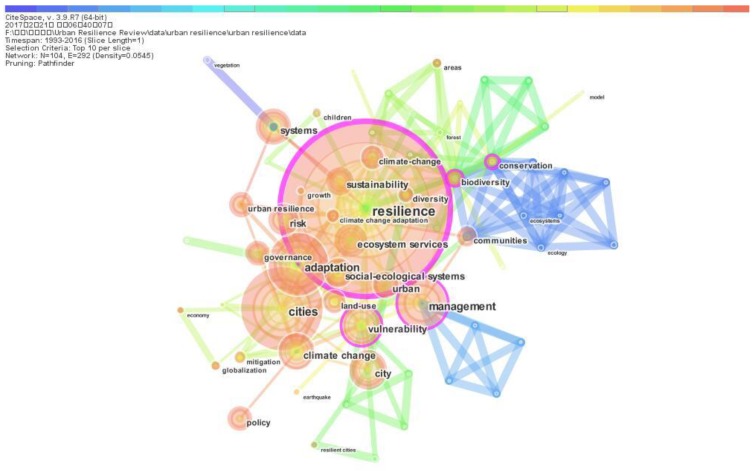
Co-occurring keywords network.

Co-occurring keyword network analysis provides the basic information of core research content and helps researchers to track the development trends of research topics at different stages of urban resilience research. In co-occurring keyword analysis process using CiteSpace, keywords are composed of “author keywords (DE)” and “keyword plus (ID)”. The author keyword (DE) is derived from the author’s article, and the keyword plus (ID) is identified by the journal which the author’s articles are published. In the process of co-occurring keywords analysis, the keywords with similar connotations are analyzed combined, such as the keywords that resilience and resiliency. Considering co-occurring keywords network with a large number of nodes, we select the first tab Top 10 per slide as the nodes selection criteria in this section. Figure 6 is the co-occurring keywords network of urban resilience research which consists of 104 nodes and 333 links. Nodes appear the shape of tree rings which have different spectrum of colors corresponding to the years of keywords’ occurring. The links also represent different colors according to the years that two keywords occurring together.

Figure 6 shows that “city OR cities OR urban’’ with a frequency of 112 is the most important keywords. Other keywords with high frequency are “resilience’’ (111), “adaptation’’ (65), “climate change” (62), “management” (41), “biodiversity” (35), “vulnerability” (33), “systems” (28), “ecosystem services” (28), “communities” (28), “sustainability” (26), “risk” (24), “social-ecological systems” (22) and “governance” (21). The main keywords can be divided into four types. The keywords “city OR cities OR urban’’, “social-ecological systems”, “systems”, “communities” and “ecosystem services” represent the research objects of urban resilience. The keywords “resilience”, “climate change” and “risk” represent the research backgrounds of urban resilience, which means that urban resilience is mainly researched on the background of environmental uncertainty. The keywords “adaptation’’, “vulnerability” and “biodiversity” represent the research contents of urban resilience, which refer to adaptive theory, vulnerability analysis and biodiversity characteristics. The keywords “management”, “sustainability” and “governance” represent that the goals of urban resilience research are achieving sustainable development through the necessary management measures.

Some keywords with close connection relationships can be identified in Figure 6. These keywords include “resilience’’, “management”, “vulnerability”, “biodiversity” and “conservation”. Considering the betweenness centrality of nodes, the nodes of keywords with close connection relationships also have high betweenness centrality in co-occurring keywords network. The keywords “resilience’’ has the highest betweenness centrality which is 0.23, followed by “biodiversity” (0.16), “management” (0.11), “vulnerability” (0.11) and “conservation” (0.1). These keywords are the major nodes linking the urban resilience research in different phases and play important roles in the development of urban resilience research.

The keywords’ evolution trends of urban resilience research are show in Table 3. The evolution trends timeline keywords is divided into four periods according to the frequency of keywords, these four periods are 1993–2004, 2005–2010, 2011–2013 and 2014–2016. Table 3 shows the evolution trends of major focuses on urban resilience research between 1993 and 2016.

As shown in Table 3, urban resilience research mainly focused on a few topics between 1993 and 2004, such as biodiversity, environment conservation, urban systems and management measures to urban resilience. Some new topics begin to appear in urban resilience research between 2005–2010, such as adaptive theory, vulnerability analysis, ecosystem services, urbanization and land use. During 2005–2010, Resilience Alliance begins to research urban resilience from the perspective of social-ecological systems. Urban resilience started to get attention in the USA between 2011 and 2013. At the same time, researchers continue to be concerned about the topics of social-ecological systems, climate change and adaptation during this period. Each topic keep the state of prosperity between 2014–2016, management measures and biodiversity get great attentions to the urban resilience researchers with the unprecedented urbanization process and the growing number of natural disasters.

### 3.3. Co-Citation Analysis

Co-citation analysis provides a unique insight into the structure and dynamics of the scientific paradigm. As a way to measure the proximity between documents, co-citation analysis is a good way to show the relationship between source articles in a dataset and corresponding citations in a wide range of external reference records [92]. Co-citation analysis includes co-citation analysis of journals, co-citation analysis by authors, and co-citation analysis based on citations [93]. In addition to the above three co-citation analysis, cluster analysis is also used to detect and identify changes in development trends in different periods, and to clarify the relationship between important turning points and urban resilience research trends.

#### 3.3.1. Journal Co-Citation Network

The function of retrieval records analysis is used to indicate the major journals source for urban resilience research. Table 4 summarizes the analysis results of journals source, the top 11 major journals source for urban resilience research is indicated identified by 355 retrieval records from 39 journals related to urban studies in the WOS core collection database.

Table 4 shows that the top 11 journals published a total of 289 articles between 1993 and 2016, which accounts for 81.42% of the total 355 articles. The journal *Landscape and Urban Planning* has the highest number of publications and published 70 (19.72%) articles on urban resilience research. *Landscape and Urban Planning* also published the first article on urban resilience research among the 355 retrieved records from 39 journals related to urban studies in the WOS core collection database [91].

**Figure 7:**
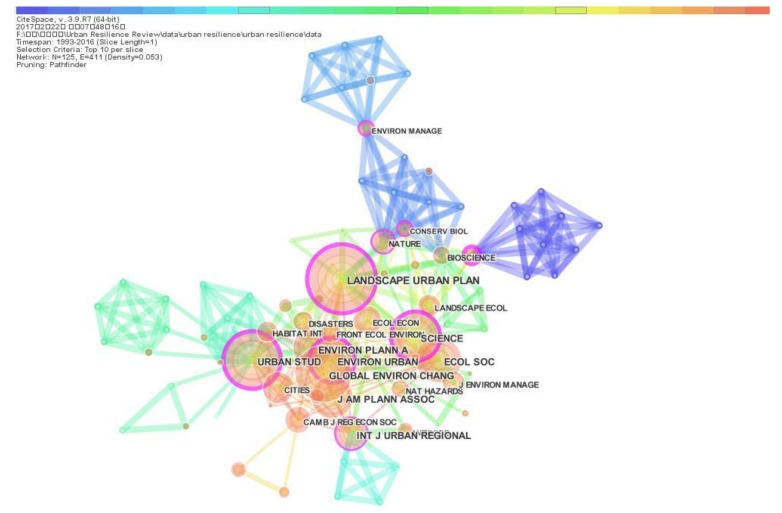
Journal co-citation network.

The 355 retrieval records cite references from different journals as the knowledge foundation of urban resilience research. In this section, CiteSpace is used to generate journal co-citation network. Considering journal co-citation network with a large number of nodes, we select the first tab Top 10 per slide as the nodes selection criteria in this section. The journal co-citation network is used to analyze and evaluate the impact and co-citation models of cited journals. The journal co-citation network is shown in Figure 7. The size of each node in journal co-citation network represents the co-citation frequency of corresponding journal. The influence of cited journals is primarily assessed by its citation frequency.

Figure 7 shows that the journal co-citation network includes 125 nodes and 437 links derived from 1084 cited journals. *Landscape and Urban Planning* is the most important cited journals with the highest cited frequency (with 100 citations), which is also the journal with the highest number of publications for urban resilience research. Other journals with high cited frequency are *Urban Studies* (with 89 citations), *Global Environmental Change* (with 83 citations), *Ecology and Society* (with 75 citations), *Science* (with 68 citations), *Journal of the American Planning Association* (with 64 citations), *Environment and Planning A* (with 63 citations), *Environment and Urbanization* (with 63 citations), *International Journal of Urban and Regional Research* (with 56 citations), *Cities* (with 49 citations), *Ecological Economics* (with 47 citations), *Nature* (with 45 citations), *Landscape Ecology* (with 40 citations) and *Habitat International* (with 40 citations).

According to the co-citation frequency, most of journals with high influence are journals from among the 39 journals related to urban studies in the WOS core collection database, including *Landscape and Urban Planning, Urban Studies, Journal of the American Planning Association, Environment and Urbanization, International Journal of Urban and Regional Research, Cities and Habitat International*. These journals all belong to the top 11 journals source listed in Table 4. However, some journals have fewer cited frequency and show lower influences than the top 11 journals listed in Table 4, such as *European Planning Studies, Urban Forestry & Urban Greening, Education and Urban Society* and *Urban Education*. *Science* and *Nature* are also journals with high influence on urban resilience research, which means that urban resilience has caused widespread concern in international academic fields. Other journals with high influence focus on general resilience research, such as *Global Environmental Change* and *Ecology and Society*.

Some journals with close connection relationships can be identified in Figure 7. These journals include *Landscape and Urban Planning, Urban Studies, Environment and Urbanization, International Journal of Urban and Regional Research* and *Nature*. These journals also have high betweenness centrality in journal co-citation network. *Environment and Urbanization* has the highest betweenness centrality which is 0.34, followed by *Urban Studies* (0.33), *Landscape and Urban Planning* (0.3), *Science* (0.22), *Biological Conservation* (0.21), *International Journal of Urban and Regional Research* (0.19), *Nature* (0.18), *Journal of Environmental Management* (0.17) and *Conservation Biology* (0.13). These journals are the major nodes as the knowledge foundation of urban resilience research in different phases. Some journals with high influence are not the core nodes in journal co-citation network, such as *Journal of the American Planning Association*.

#### 3.3.2. Author Co-Citation Network

**Figure 8:**
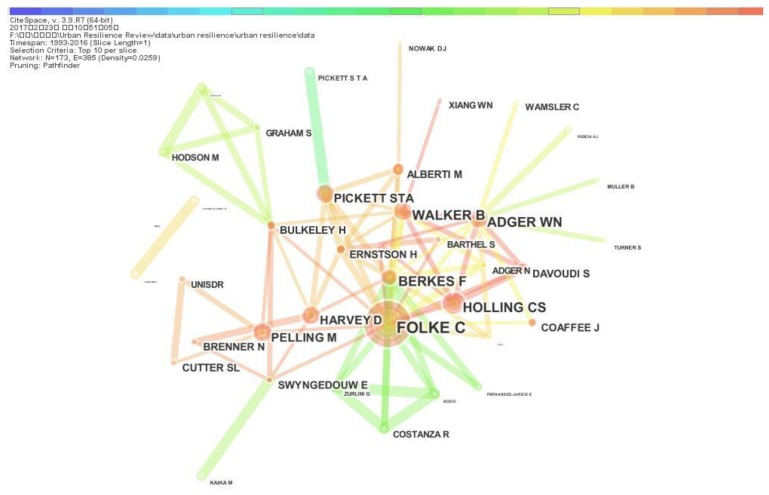
Author co-citation network.

Author co-citation analysis is an efficient method to measure relationships and connections between authors and to describe mainstream, or at least the leading edge, in the collaborative structure of urban resilience research. In this section, we identify and analyze the evolutionary trends of authors whose scientific papers are cited in the same paper and community of science. Consider a co-citation network with multiple nodes, selecting the first ten tabs of the first slide as the node selection criteria in this section. The author co-citation network is shown in Figure 8, which contains 173 author nodes and 412 co-citation links. In author co-citation network, the size of each node reflects co-citation frequency between authors, and the link represents an indirect collaborative relationship between authors. Some scientific communities have significant co-citation relationships in author co-citation network.

According to the analysis results of author co-citation network, we identify and analysis the authors with highly cited frequency and their countries source. Figure 8 shows that the author with the highest cited frequency is Carl Folke (52 citations, Sweden), followed by Crawford Stanley Holling (40 citations, Canada and USA), Brian Walker (37 citations, Australia), William Neil Adger (35 citations, UK), Fikret Berkes (35 citations, Canada), Mark Pelling (33 citations, UK), Steward T.A. Pickett (32 citations, USA), David Harvey (25 citations, USA), World Bank (22 citations, UN), Susan L. Cutter (18 citations, USA), Simin Davoudi (18 citations, UK), Henrik Ernstson (18 citations, Sweden), Andy Pike (18 citations, UK), Saskia Sassen (16 citations, USA), Philip R. Berke (16 citations, USA), Harriet Bulkeley (16 citations, UK), Jon Coaffee (16 citations, UK), Erik Swyngedouw (15 citations, UK), Marina Alberti (14 citations, USA) and Neil Brenner (14 citations, USA). In addition to the World Bank, other top 20 authors with highly cited frequency are natural person. These 19 authors all come from the developed countries including USA (8), UK (7), Sweden (2), Canada (2) and Australia (1). The distribution of highly cited authors’ countries indicates that urban resilience research has become a hot topic in major countries around the world.

As the highest cited author, Carl Folke, Professor at the Stockholm Resilience Centre at Stockholm University, focuses on the role that social-ecological systems at different scales play in social and economic development, and how to govern and manage integrated social-ecological systems towards resilience [94]. Henrik Ernstson also comes from Stockholm University and studies resilience under the background of climate change [33], which manifests that Stockholm University is an important research center for resilience. Crawford Stanley Holling is the pioneer of resilience science and first proposed the concept of resilience in the social and ecology domain [1]. Urban resilience researchers hold the largest proportion of top 20 authors with highly cited frequency (6/20); these authors include William Neil Adger, David Harvey, Philip R. Berke, Jon Coaffee, Erik Swyngedouw and Marina Alberti.

#### 3.3.3. Document Co-Citation Network

Document co-citation analysis is used to measure the dependency relationships of previous urban resilience research. The document co-citation analysis assumes that the two documents cited in the same article are similar from a theoretical point of view. Documents with similarities are commonly cited together, and the similarities of these documents depend on the frequency of co-citations. This method reveals the structure of academic research and predicts future research directions. Clusters are generated during the process of document co-citation analysis and used for evolution trends analysis.

In the process of generating a document co-citation network, the node selection criteria include three sets of thresholds: citation threshold (c), co-citation threshold (cc), and co-citation coefficient threshold (ccv). Referencing to previous research on document co-citation by Chen in 2006 [80], these three sets of threshold levels are set as follows: (2, 1, 10), (3, 1, 0), and (3, 2, 10). The time span of 24-years between 1993 and 2016 is divided into twenty four 1-year time slices. Table 5 summarizes the construction configuration for document co-citation network.

There is no article related urban resilience research in three 1-year slices of this study dataset, these three 1-year slices are 1994–1994, 1998–1998 and 1999–1999. Thus, no co-citation document exists in above three 1-year slices. Document co-citation network begins to appear the network nodes and links together after 2000. The numbers of nodes and links in the last four years account for the most of total nodes and links’ numbers, which manifests that the document co-citation network in this study can reflects the emerging development trends of urban resilience research.

**Figure 9:**
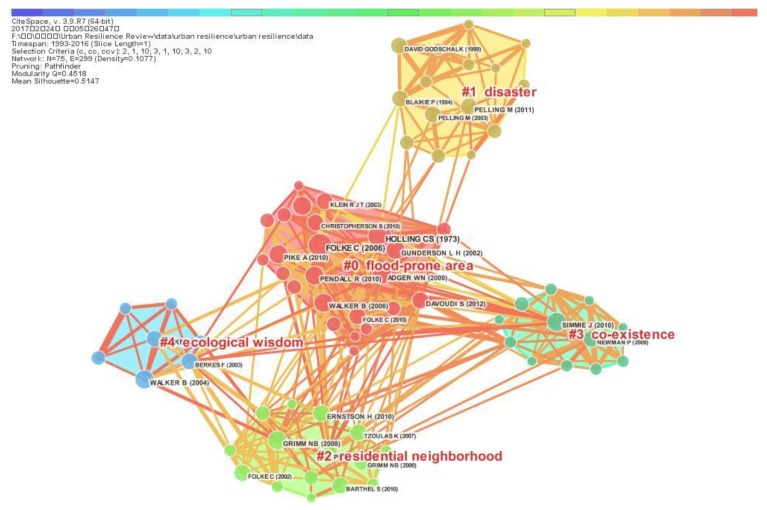
Cluster view of document co-citation network.

This study generates the document co-citation network based on 17,283 references cited by 355 retrieval records. The clusters of document co-citation network are identified by labels and extract noun phrases from the title terms (T) of documents. Each node represents a document for urban resilience studies, with the label showing the author’s name and year of publication. Each link between nodes reflects the co-citation relationships of two documents in the same article. The document co-citation network is shown in Figure 9 and includes 73 documents’ nodes and 306 links. Figure 9 illustrates this document co-citation network comprising five representative clusters with closely located nodes. The labels for these five clusters are generated by the inverted document frequency (tf * idf) algorithm. The modularity Q value of 0.4518 is close to 0.5, which means that the division of clusters is relatively reasonable and can be accepted. The average silhouette value is 0.5147, which indicates that the homogeneity of these clusters is at a reasonable level.

In Figure 9, the document co-citation network is divided into different clusters which are described by different colors. The nodes with close co-citation relationship are marked the same color and concentrated in one cluster which has the same background color as the nodes. The size of the cluster represents the number of nodes in each cluster. As shown in Figure 9, cluster #0 is larger than other clusters and has 24 nodes, and there is a close co-occurrence relationship between the nodes of cluster #0 in 2005. The links in cluster #0 have four kinds of colors from light yellow to deep red, which indicates that the nodes of cluster #0 close co-citation relationships between 2011 and 2016. The clusters #1 and #2 have 15 numbers of nodes and forms in 2005 and 2006. The newest cluster is cluster #3 with 12 nodes which forms in 2007. Although the cluster #4 forms in 2003, it only contains 7 nodes. Cluster #1 has the most connections with clusters #2 and #3. The most frequently referenced document in cluster #0 is Folke (2006)#1, which connects cluster #0 and cluster #1 2, #3 and #4 [94].

We used CiteSpace to analyze 17,283 references cited in 355 retrieval records from 39 journals related to urban studies in WOS core collection database city. Table 6 summarizes the top 19 commonly cited references. In the process of selecting top 19 co-cited documents, we first select 21 documents that have been co-cited more than 10 times. Three documents of between centrality close to 0 are deleted from high co-cited documents. Another document with high betweenness centrality is also selected as high co-cited document. Finally, this study selects 19 documents as high co-cited documents. Table 4 provides detailed information of the top 19 co-cited documents.

The top 19 co-cited documents is presented in Table 4 can be divided into four topics: the basic concepts and theories of resilience (8/19), the conceptual models and pathways of urban resilience (6/19), the adaptive capacity of social-ecological systems (3/19) and resilience analysis in the scenarios of climate change and natural hazards (2/19). As the research foundation of urban resilience, the documents related to the basic concepts and theories of resilience accounts for the largest proportion of top 19 co-cited documents. All these 8 documents belong to cluster #0 which includes No. 1, No. 2, No. 5, No. 6, No. 8, No. 10, No. 11 and No. 15. The concept of “resilience” is first proposed in the social and ecology domain [1]. Human-natural systems are abstracted into a theoretical resilience model used to understand some methods that can help researchers develop effective policies for environmental management [95,96]. The resilience approach emphasizes nonlinear dynamics, thresholds and uncertainties, and focus on the effects of dynamic interactions on human-natural systems at spatiotemporal scales [94,97]. As a valuable concept for climate change, resilience is applied to the planning theory and practice of human society and urban society [16,98,99].

Cluster #2 and cluster #3 are combined as the top co-cited documents focuses on the conceptual models and pathways of urban resilience. Urban resilience is a promising new tool to promote further urban design and proposes a conceptual framework for promoting the sustainable development of urban ecosystems and human health [100,101]. Under the background of climate change and energy shortages, Cities present various problems and resilience provides a new way to above challenges in increasing urbanization world [33,102]. Transition is an important pathway of urban resilience and should be considered as an evolutionary approach [103,104]. Cluster #4 consists of 3 top co-cited documents relating to the adaptive capacity of social-ecological systems which strongly focus on the thresholds of change and upgrading the adaptive capacity by appropriate strategies [95,96,97,98,99,100,101,102,103,104,105,106,107]. Cluster #1 only includes 2 top co-cited documents which refer to resilience analysis in the scenarios of climate change and natural hazards; resilience is an important transformation to protect core assets or functions from the risks of climate change [108,109].

### 3.4. Evolution Trends Analysis

In this section, we use representative documents in document co-citation network to identify the main research topics in each cluster. The main research topics in each cluster represent the key focuses of urban resilience research. The academic structure of urban resilience research is described based on the analysis of the main research topics of each cluster, including the relationship between the research content of different clusters. Table 7 summarizes five document co-citation clusters by document co-citation analysis. Each row of Table 7 contains cluster ID, size, silhouette value, cluster label (TFIDF), representative documents in the cluster, the most representative citing document and cluster label (MI). The size of cluster represents the number of co-cited references, and the representative cited references reflect the main research topics of cluster. The cluster label (TFIDF) is the label for the different components in each cluster, and the cluster label (MI) is the combined label for each cluster.

As the largest cluster, Cluster #0 contains 24 documents in document co-citation network, its silhouette value is 0.609. Cluster #0 is labeled as “flood-prone area”, the cluster label (MI) is combined as “resilience exploratory analysis” through reviewing relevant citing and co-cited documents. This cluster focuses on the basic theories and methods of resilience; the concept is applied to urban planning and land use analysis. To understand the dynamics of social development, the resilience perspective is increasingly used to develop the adaptive management approach for responding to regional change [89,94]. The most representative document in cluster #0 is Schmidt and Garland [110], which define resilience as the ability to reduce vulnerability and addresses how resilience thinking can assist planners and their communities in disaster-prone regions.

Cluster #1 is the second largest cluster, which contains 15 documents in document co-citation network, its silhouette value is 0.924. Cluster #1 is labeled as “disaster”, the cluster label (MI) is combined as “disaster resilience” through reviewing relevant citing and co-cited documents. The top 2 representative documents in cluster #1 are Blaikie et al. [108] and Pelling [109]. These researchers analyzed the impact of climate change on human development and used the concept of resilience to guide human society’s vulnerability to natural disasters. The most representative document in cluster #1 is Nguyen and Salvesen, which reveals that culture is essential for an effective disaster recovery process and more appropriate and effective disaster recovery plans can be developed by analyzing resilience prior to a disaster [111].

Cluster #2 contains 15 documents in its document co-citation network; its silhouette value is 0.507. Cluster #2 is labeled as “residential neighborhood”, the cluster label (MI) is combined as “urban resilience” through reviewing relevant citing and co-cited documents. The most representative documents in cluster #2 are published by Bolunda and Hunhammar [112] and Pickett et al. [113]. These researchers have proposed an emerging urban resilience framework that considers ecosystem services having significant impacts on the quality of life in urban areas. The representative document in cluster #2 is Hunter and Brown [114], in which the potential value of social contagion has been discussed as a mechanism to expand sustainable behavior that supports ecological resilience in urban areas.

Cluster #3 contains 12 documents in document co-citation network, its silhouette value is 0.736. It is labeled as “co-existence”, the cluster label (MI) is combined as “urban resilience practice”. The most representative documents in cluster #3 are published by Simmie and Martin [104] and Evans [115]. These researchers focus on adaptive cycle theory and make assumptions about urban and regional resilience. The representative citing document of cluster #3 is that of Ozuduru et al. [116], which think that the sustainability of cities depends on the viability of city center and the resilience of shopping district.

Cluster #4 containins 7 documents in document co-citation network, its silhouette value is 0.827. It is labeled as “ecological wisdom”, the cluster label (MI) is combined as “social-ecological systems”. The most representative documents in cluster #4 are published by Berkes et al. [105] and Folke et al. [107]. These documents research urban resilience from the perspective of social-ecological systems and investigate the reorganization of urban systems during periods of abrupt change. The representative citing document of cluster #4 is that of Patten [117], in which ecological wisdom as a comprehensive management approach play an important role in creating sustainable and resilient urban and natural ecosystems.

To explore the evolution trends of urban resilience research, some important documents with strong citation bursts are selected from the document co-citation network. These documents provide high potential guides for future urban resilience research. The development and evolution trends of urban resilience research are identified by analyzing these important documents with strong citation bursts. Future directions for urban resilience research can be forecasted and further explored by analyzing the existing major research topics. We think that the topics with significant increase in a short time will represent a developing trend in future. This study selects documents and topics with strong citation bursts in recent years to reflect the newest trends of urban resilience research. We used CiteSpace to generate a summary list of documents associated with the referenced burst. Table 8 shows which document has the strongest reference burst and which period has the strongest burst occurrence.

Table 8 summarizes the information of top seven references with strongest citation bursts. According to the begin year, all bursts are generated after 2010, which manifests that urban resilience has only become hot topics recently. From 2011 to 2016, urban resilience research focuses on the definition of urban resilience [1,16,94], the adaptation of urban resilience [109,118], urban resilience model [16,100], the case studies of urban resilience [100,119], urban resilience and vulnerability analysis [109] and urban social-ecological systems [1,94].

## 4. Discussion

Urban resilience gained great popularity in both academia and industry with the unprecedented urbanization process. Great efforts have been made to enhance urban resilience in the process of urbanization. The concept of resilience has been applied to enhance cities’ ability to resist disasters. Urban resilience is an important research topic in urban resilience research. Meerow et al. summarized different definitions from 25 previous publications, the results manifest that urban resilience is an inconsistent and ambiguous concept [10]. Because numerous disciplines engage in the field of urban resilience research, Da Silva et al. think that there are great challenges associated with defining and characterizing “urban” and “resilience” separately [28]. These conceptual inconsistencies make it difficult to apply some resilience metrics to many scenarios. The results of this study also show that the definition of urban resilience is still an important research topic in future urban resilience research. A common definition of urban resilience should be proposed in future urban resilience research. Some scholars provide a common definition of urban resilience. Meerow et al. for instance, defines urban resilience as the ability of an urban system and its constituent socio-ecological and socio-technical networks across temporal and spatial scales to maintain or rapidly return to desired functions in the face of a disturbance, to adapt to changes, and to quickly transform systems that limit current or future adaptive capacity [10].

Scientometric analysis becomes an important research method for reviewing research status in different academic domains. Scientometric methods include qualitative, quantitative and computational approaches, which focus on institutional productivity comparisons, institutional research rankings, journal rankings establishing faculty productivity and tenure standards, assessing the influence of top scholarly articles, and developing profiles of top authors and institutions in terms of research performance [72,73,74,75]. Previous similar studies mainly use a single scientometric indicator to analyze the research status of a discipline [76]. These research results only reflect the research status of the discipline from one specific aspect, such as co-authors analysis only analyze authors and their institutions, research social structures and collaborative networks [77]. Thus, the research results of single scientometric indicator cannot reveal the research status of a discipline. This study adopts three types of scientometric indicators, including co-author analysis, co-word analysis and co-citation analysis, which show influential authors, primary countries and research institutions, core categories and topics of focus, core journals, and evolution trends on urban resilience research. The research results reveal the emerging evolutionary trends and a sufficiently large and high-quality body of research that accurately reflects the global picture of urban resilience research.

Co-citation analysis is used to analyze the academic structure of urban resilience in previous studies. Fröhlich and Hassink use the page rank indicator to analyze the citation network of regional resilience, and the results manifest that “Urban Ecology and Policies” is the largest community in co-citation network [66]. Resilience on city-scales is the most important research topic which concerns environmental shocks and hazards, as well as policy about urban or regional governance, as well as resilience on an individual level with a focus on psychology and society. Fröhlich and Hassink identify common keywords, including “climate change/risks/disaster(s), governance/community/social capital”, as well as “adaptive capacity/regional adaptation/sustainability”, but do not give the applicable fields of these keywords. In the section “Network of Co-occurring Keywords”, we divide the keywords of urban resilience research into four types and analyze the connotations of these keywords. The keywords “city OR cities OR urban’’, “social-ecological systems”, “systems”, “communities” and “ecosystem services” represent the research objects of urban resilience. The keywords “resilience”, “climate change” and “risk” represent the research backgrounds of urban resilience, suggesting that urban resilience mainly studies the background of environmental uncertainty. The keywords “adaptation’’, “vulnerability” and “biodiversity” represent the research contents of urban resilience, which refer to adaptive theory, vulnerability analysis and biodiversity characteristics. The keywords “management”, “sustainability” and “governance” represent that the goal of urban resilience research is to achieve sustainable development by taking necessary management measures.

Fröhlich and Hassink offer five key urban resilience documents by qualitative analysis, these five key documents include Pike et al. [98], Christopherson et al. [120], Hassink [121], Pendall et al. [99] and Folke [94]. We summarize the top 19 co-cited documents in the document co-citation network of urban resilience. These top 19 co-cited documents already include the documents Pike et al. [98], Pendall et al. [99] and Folke [94]. The documents Christopherson et al. [120] and Hassink [121] present a significant regional resilience perspective and do not be included in top 19 co-cited documents. We divide the top 19 co-cited documents into four topics: the basic concepts and theories of resilience (8/19), the conceptual models and pathways of urban resilience (6/19), the adaptive capacity of social-ecological systems (3/19) and resilience analysis in the scenarios of climate change and natural hazards (2/19). Compared with Fröhlich and Hassink’s paper, the most significant contribution of this study is to reveal the latest trends of urban resilience research. This study summarizes the top seven documents with strong citation dramatic increase between 2011 and 2016. These seven documents reflect the newest trends of urban resilience: the definition of urban resilience, the adaptation of urban resilience, urban resilience model, the case studies of urban resilience, urban resilience and vulnerability analysis and urban social-ecological systems.

## 5. Conclusions

Urban resilience has become a very popular area of research among academics and practitioners with the unprecedented urbanization. Great efforts have been made to enhance urban resilience in the process of urbanization. This study explores the emerging trends of urban resilience research by scientometric analysis from 1993 to 2016. Some highlights in this study are listed as follows:

(1) Identifying the lead authors with significant contributions and influences based on co-authorship and author co-citation analysis. The results indicate that authors with a large number of publications do not necessarily have significant impacts on urban resilience research. Instead, some less productive authors maybe have larger influences on urban resilience research.
(2) Discovering the evolutionary trend of geographic distribution on urban resilience research by analyzing co-authors’ countries. The result shows that urban resilience research is conducted mainly in countries with high urbanization levels or in the process of urbanization (e.g., USA, England, Australia, Canada, China and Sweden). As the biggest country in the process of urbanization, China has close collaborative relationships with most countries of high publications output, including the US, England, Australia and Canada.
(3) Analyzing the evolution trend of research hotspots on urban resilience by co-word analysis. The analysis results show that the research popular areas of urban resilience have been continuously shifting from 1993 to 2016. Top 20 keywords, indicators of the transformation of important research topics in urban resilience, have changed tremendously.
(4) Providing the preferred journals according to the journal co-citation analysis. The results identified leading journals in urban resilience research. The results manifest these journals with lot of publications also have bigger influences on urban resilience research, such as *Environment and Urbanization, Urban Studies* and *Landscape and Urban Planning*.
(5) Discussing the emerging research trends of urban resilience by document co-citation analysis. The results show that document co-citation network includes five typical regarding following themes: the basic concepts and theories of resilience, the conceptual models and pathways of urban resilience, the adaptive capacity of social-ecological systems, and resilience analysis in the scenarios of climate change and natural hazards. The emerging research trends are summarized by representative documents references with greatest citation increase in each cluster. These emerging research trends in urban resilience contain urban resilience definition, urban resilience adaptation model, case studies for urban resilience, urban resilience analysis methods and urban social-ecological systems.

This study offers a valuable guide for the future research in urban resilience. The analysis results provide influential authors, primary countries and research institutions, core categories and topics of focus, core journals, and evolution trends on urban resilience research. This study analyzes emerging evolution trends of urban resilience research by scientometric analysis, which reflect a sufficiently large and high-quality body of research that accurately reflects the whole picture of urban resilience research. The results also reveal important highlights and future emerging research trends of urban resilience, which will help researchers for future collaboration and work.

## Figures and Tables

**Table 1:** The publication frequencies, institutions of the top 13 most productive authors.

Frequency	Year	Author	Institution
4	2014	Wesley E. Highfield	Texas A&M University
4	2010	Debra Roberts	EThekwini Municipal
3	2014	Shannon Van Zandt	Texas A&M University
3	2014	Walter Gillis Peacock	Texas A&M University
3	2011	Robert F. Young	University of Texas at Austin
3	2010	Cassidy Johnson	UCL
3	2010	Huraera Jabeen	Population Council

Note: Year is the latest year that the author published papers, UCL: University College London.

**Table 2:** The subject categories of 355 publications on urban resilience research.

Category	1993–1999	2000–2010	2010–2016	1993–2016	%
Urban Studies	4	67	284	355	100
Environmental Studies	3	39	177	219	61.69
Geography	3	24	85	112	31.55
Planning Development	0	20	75	95	26.76
Geography Physical	3	14	53	70	19.72
Ecology	3	14	53	70	19.72
Education & Educational Research	1	10	12	23	6.48
Plant Science	0	1	12	13	3.66
Forestry	0	1	12	13	3.66
Architecture	0	2	3	5	1.41
History of Social Science	0	1	3	4	1.13
History	0	1	3	4	1.13
Engineering Civil	0	0	4	4	1.13
Economics	0	1	2	3	0.85
Law	0	0	2	2	0.56
Sociology	0	0	1	1	0.28

**Table 3:** The evolution trends of top 20 keywords on urban resilience research.

Category	Frequency				
	1993–2004	2005–2010	2011–2013	2014–2016	1993–2016
city	1	11	40	60	112
resilience	1	11	30	69	111
adaptation	0	7	21	37	65
climate change	0	9	21	32	62
management	2	4	9	26	41
biodiversity	2	5	8	20	35
vulnerability	0	7	10	16	33
systems	2	4	7	15	28
ecosystem services	0	2	10	16	28
communities	1	3	8	16	28
sustainability	0	2	10	14	26
risk	0	5	6	13	24
social-ecological systems	0	3	12	7	22
governance	0	2	6	13	21
policy	0	1	2	16	19
urbanization	0	3	7	9	19
perspective	1	0	8	6	15
land use	0	2	3	9	14
conservation	2	4	4	3	13
united states	0	0	5	6	11

**Table 4:** The top 11 journals source for urban resilience research according to the 355 publications retrieved from the WOS core collection database.

Journal	Number	%
Landscape and Urban Planning	70	19.72
Environment and Urbanization	41	11.55
Cities	34	9.58
Habitat International	28	7.89
Urban Studies	28	7.89
International Journal of Urban and Regional Research	20	5.63
European Planning Studies	17	4.79
Journal of the American Planning Association	15	4.23
Urban Forestry & Urban Greening	13	3.66
Education and Urban Society	12	3.38
Urban Education	11	3.1

**Table 5:** The construction configuration for 73 documents co-citation network.

1-Year Slice	c	cc	ccv	Number		
				Documents	Nodes	Links
January 1993–December 1993	2	1	0.1	23	0	0
January 1994–December 1994	2	1	0.09	0	0	0
January 1995–December 1995	2	1	0.08	96	0	0
January 1996–December 1996	2	1	0.08	9	0	0
January 1997–December 1997	2	1	0.07	31	0	0
January 1998–December 1998	2	1	0.06	0	0	0
January 1999–December 1999	2	1	0.05	0	0	0
January 2000–December 2000	2	1	0.04	81	0	0
January 2001–December 2002	2	1	0.03	75	0	0
January 2002–December 2002	2	1	0.03	79	0	0
January 2003–December 2003	2	1	0.02	58	0	0
January 2004–December 2004	2	1	0.01	264	1	0
January 2005–December 2005	3	1	0	148	0	0
January 2006–December 2006	3	1	0.01	253	0	0
January 2007–December 2007	3	1	0.02	329	0	0
January 2008–December 2008	3	1	0.03	385	0	0
January 2009–December 2009	3	1	0.04	653	0	0
January 2010–December 2010	3	1	0.05	715	0	0
January 2011–December 2011	3	1	0.05	1054	2	1
January 2012–December 2013	3	1	0.06	1810	5	8
January 2013–December 2013	3	1	0.07	2645	24	79
January 2014–December 2014	3	1	0.08	3301	29	81
January 2015–December 2015	3	1	0.09	3591	29	81
January 2015–December 2016	3	2	0.1	3196	21	65
Total				18,796	110 (73)	315 (306)

**Table 6:** The top 19 co-cited documents in document co-citation network.

No.	Co-Citation Frequency	Between Centrality	Author	Year	Title	Source	Type	Cluster
1	31	0.31	Carl Folke	2006	Resilience: the emergence of a perspective for social-ecological systems analyses	*Global Environmental Change*	article	#0
2	29	0.4	Crawford Stanley Holling	1973	Resilience and stability of ecological systems	*Annual Review of Ecology and Systematics*	article	#0
3	19	0.12	Steward TA Pickett et al.	2004	Resilient cities: meaning, models, and metaphor for integrating the ecological, socio-economic, and planning realms	*Landscape and Urban Planning*	article	#2
4	18	0.14	James Simmie and Ron Martin	2010	The economic resilience of regions: towards an evolutionary approach	*Cambridge Journal of Regions, Economy and Society*	article	#3
5	17	0.01	Andy Pike et al.	2010	Resilience, adaptation and adaptability	*Cambridge Journal of Regions, Economy and Society*	article	#0
6	16	0.05	Brain Waller and David Salt	2006	Resilience thinking	*Island Press*	book	#0
7	15	0.07	Brian Walker and Jacqueline A. Meyers	2004	Thresholds in Ecological and social–ecological systems: a developing database	*Ecology and Society*	article	#4
8	15	0.01	William Neil Adger	2000	Social and ecological resilience: are they related?	*Progress in Human Geography*	article	#0
9	15	0.06	Nancy B. Grimm et al.	2008	Global change and the ecology of cities	*Science*	article	#2
10	14	0.1	Rolf Pendall et al.	2010	Resilience and regions: building understanding of the metaphor	*Cambridge Journal of Regions, Economy and Society*	article	#0
11	14	0.01	Lance H. Gunderson and C.S. Holling	2002	Panarchy: understanding transformations in human and natural systems	*Island Press*	book	#0
12	13	0.05	Henrik Ernstson et al.	2010	Urban transitions: on urban resilience and human-dominated ecosystems	*AMBIO*	article	#2
13	13	0.06	Mark Pelling	2011	Adaptation to climate change: from resilience to transformation	*Routledge*	book	#1
14	13	0.06	Carl Folke et al.	2005	Adaptive governance of social-ecological systems	*Annual Review of Environment and Resources*	article	#4
15	13	0.31	Simin Davoudi et al.	2012	Resilience: a bridging concept or a dead end?	*Planning Theory and Practice*	article	#0
16	12	0.09	Fikret Berkes et al.	2003	Navigating social-ecological systems: building resilience for complexity and change	*Cambridge University Press*	book	#4
17	12	0.06	Peter Newman et al.	2009	Resilient cities: responding to peak oil and climate	*Island Press*	book	#3
18	11	0.01	Konstantinos Tzoulas et al.	2007	Promoting ecosystem and human health in urban areas using green infrastructure: a literature review	*Landscape and Urban Planning*	article	#2
19	10	0.29	Piers Blaikie et al.	1994	At risk: natural hazards, people’s vulnerability and disasters	*Routledge*	book	#1

**Table 7:** The 5 clusters’ contents in document co-citation network.

ID	Size	Silhouette	Label (TFIDF)	Representative documents in Cluster	The most representative citing document	Label (MI)
#0	24	0.609	(6.66) flood-prone area; (5.9) texa; (4.73) land use; (4.73) urban wildscape; (4.73) mombasa	Carl Folke (2006) “Resilience: The emergence of a perspective for social–ecological systems analyses” Rolf Pendall et al. (2010) “Resilience and regions: building understanding of the metaphor”	Deanna Harlene Schmidt and Kathleen A. Garland (2012) “Bone dry in texas: resilience to drought on the upper texas gulf coast” (0.17)	resilience exploratory analysis
#1	15	0.924	(9.49) disaster; (6.66) vulnerability; (5.86) disaster recovery; (5.86) evolution; (5.86) resiliency	Mark Pelling (2011) “Adaptation to Climate Change: From Resilience to Transformation” Piers Blaikie et al. (1994) “At Risk: Natural Hazards, People’s Vulnerability and Disasters”	Mai Thi Nguyen and David Salvesen (2014) “Disaster recovery among multiethnic immigrants: a case study of southeast asians in bayou la batre (al) after hurricane katrina” (0.13)	disaster resilience
#2	15	0.507	(5.86) residential neighborhood; (5.86) gardening; (5.86) green infrastructure; (5.86) ecology; (5.86) spatial contagion	Per Bolunda and Sven Hunhammar (1999) “Ecosystem services in urban areas” Steward T. A. Pickett et al. (2008) “Beyond Urban Legends: An Emerging Framework of Urban Ecology, as Illustrated by the Baltimore Ecosystem Study”	Mary Carol R. Hunter and Daniel G. Brown (2012) “Spatial contagion: gardening along the street in residential neighborhoods” (0.2)	urban resilience
#3	12	0.736	(4.73) co-existence; (4.73) arizona; (4.73) shopping venue; (4.73) urban retail system; (4.73) resilience assessment	James Simmie and Ron Martin (2010) “The economic resilience of regions: towards an evolutionary approach” J P Evans (2011) “Resilience, ecology and adaptation in the experimental city”	Burcu H. Ozuduru et al. (2014) “Do shopping centers abate the resilience of shopping streets? the co-existence of both shopping venues in ankara, turkey” (0.17)	urban resilience practice
#4	7	0.827	(8.6) ecological wisdom; (5) design; (4.73) modernity; (4.73) emerging field; (4.73) natural ecosystem	Fikret Berkes et al. (2003) “Navigating Social-Ecological Systems” Carl Folke et al. (2005) “Adaptive governance of social-ecological systems”	Duncan T. Patten (2016) “The role of ecological wisdom in managing for sustainable interdependent urban and natural ecosystems” (0.29)	social-ecological systems

Note: TFIDF is the label of different units in each cluster; MI is the combined label for each cluster.

**Table 8:** The top 7 references with strongest citation bursts.

References	Strength	Begin Year	End Year
Folke. (2006), Global environ chang, V16, P253 [94]	3.8259	2001	2016
Pickett. (2004), Landscape urban plan, V69, P369 [100]	3.5415	2013	2014
Pelling. (2011), Adaptation to climate change: from resilience to transformation, V, P1 [109]	3.3859	2013	2016
Davoudi. (2012), Planning theory prac, V13, P299 [16]	3.3321	2013	2016
Folke. (2010), ECOL SOC, V15, P [118]	4.3471	2015	2016
Vale. (2005), Resilient cuty moder, V, P [119]	3.3199	2015	2016
Holling. (1973), Annual rev ecol syst, V4, P1 [1]	3.3163	2015	2016
